# Hydrophobic Silk Fibroin–Agarose Composite Aerogel Fibers with Elasticity for Thermal Insulation Applications

**DOI:** 10.3390/gels10040266

**Published:** 2024-04-15

**Authors:** Yuxiang Du, Pengjie Jiang, Xin Yang, Rui Fu, Lipeng Liu, Changqing Miao, Yaxiong Wang, Huazheng Sai

**Affiliations:** 1 School of Chemistry and Chemical Engineering, Inner Mongolia University of Science and Technology, Baotou 014010, China; duyuxiang5520@163.com (Y.D.); jpj1692787089@163.com (P.J.); yangxin975@163.com (X.Y.); lipengliu1998@163.com (L.L.); qingmc@163.com (C.M.); wangyaxiong2021@126.com (Y.W.); 2 Aerogel Functional Nanomaterials Laboratory, Inner Mongolia University of Science and Technology, Baotou 014010, China

**Keywords:** silk fibroin, agarose, aerogel fibers, mechanical properties, thermal insulation

## Abstract

Aerogel fibers, characterized by their ultra-low density and ultra-low thermal conductivity, are an ideal candidate for personal thermal management as they hold the potential to effectively reduce the energy consumption of room heating and significantly contribute to energy conservation. However, most aerogel fibers have weak mechanical properties or require complex manufacturing processes. In this study, simple continuous silk fibroin–agarose composite aerogel fibers (SCAFs) were prepared by mixing agarose with silk fibroin through wet spinning and rapid gelation, followed by solvent replacement and supercritical carbon dioxide treatment. Among them, the rapid gelation of the SCAFs was achieved using agarose physical methods with heat-reversible gel properties, simplifying the preparation process. Hydrophobic silk fibroin–agarose composite aerogel fibers (HSCAFs) were prepared using a simple chemical vapor deposition (CVD) method. After CVD, the HSCAFs’ gel skeletons were uniformly coated with a silica layer containing methyl groups, endowing them with outstanding radial elasticity. Moreover, the HSCAFs exhibited low density (≤0.153 g/cm^3^), a large specific surface area (≥254.0 m^2^/g), high porosity (91.1–94.7%), and excellent hydrophobicity (a water contact angle of 136.8°). More importantly, they showed excellent thermal insulation performance in low-temperature (−60 °C) or high-temperature (140 °C) environments. The designed HSCAFs may provide a new approach for the preparation of high-performance aerogel fibers for personal thermal management.

## 1. Introduction

Aerogels are three-dimensional nanoporous materials with low density (~0.003–0.5 g/cm^3^), high porosity (80%~99.8%), a high specific surface area (500–1200 m^2^/g), and low thermal conductivity (~15 mW m^−1^ K^−1^) [1,2,3]. Benefiting from these excellent characteristics, aerogels have broad application prospects in the fields of thermal insulation [4,5], adsorption [2,6], air purification [7,8], catalysts and catalytic carriers [9], biosensors [10], aerospace [11], etc. [12]. To date, researchers have developed a variety of aerogels, ranging from the original inorganic oxide (silica, alumina, zirconia, and titanium oxide) aerogels [13] to organic polymer (resorcinol–formaldehyde, polyvinyl chloride, and polyimide) aerogels [14], metal (Au, Pt, Ag, and Cu) aerogels [15], carbon-based (carbon nanotubes and graphene) aerogels [16], and biopolymer (cellulose [17], agarose [1], chitosan [18], sodium alginate [19], silk fibroin [20], and lignin [21]) aerogels. Among them, inorganic oxide aerogels are among the earliest and most promising aerogel materials. However, the mechanical properties of traditional inorganic oxide aerogels are poor, limiting their practical applications [22]. Although some organic polymer aerogels have excellent mechanical properties, their raw materials are non-renewable and non-degradable petroleum-based polymers, contributing to the shortage of non-renewable resources and environmental pollution. Therefore, researchers have been studying and developing renewable and degradable biopolymer aerogels.

Silk is a natural protein fiber formed by solidifying the silk liquid secreted by mature silkworms during cocooning. With its unique properties, it plays an indispensable role in the textile industry, especially in high-end clothing, functional fibers, and textiles [23]. Silk fibroin (SF) is the main component of silk, which has the advantages of sustainability, easy modification, and tissue regeneration, making it popular in the field of developing aerogels [24]. Recently, the application of aerogel materials as functional wearable materials has received considerable attention [25,26,27,28,29,30]. Among them, for example, Liu et al. [26] assembled cellulose nanofibers into super-tough nanopore aerogel fibers with a high specific surface area (372 m^2^/g), good mechanical strength (30 MPa) and ultra-high toughness (21.85 MJ/m^3^). Chen et al. [25] continuously prepared holocellulose nanofibrils/cellulose aerogel fibers with a high specific surface area (413 m^2^/g) and high strength (20.8 MPa) by using a nano-hybrid strategy and a wet spinning method. Therefore, it was particularly attractive to process SF into flexible one-dimensional aerogel fibers for the preparation of wearable thermal insulation materials and the realization of effective personal thermal management. In view of the difficulty in preparing pure SF aerogel fibers and their poor mechanical properties, a common approach has been to composite SF with other materials. Inspired by the structure of polar bear hair, Bai et al. [31] realized the continuous and large-scale preparation of porous SF aerogel fibers with an aligned porous structure based on the freeze-spinning strategy, and the woven fabrics from them have excellent thermal insulation performance. However, the SF aerogel fibers, known for their highly porous structure, have poor mechanical properties, and their maximum tensile strength and elongation at break were only 0.95 MPa and 0.080%, respectively. Moreover, Mitropoulos et al. [32] prepared SF/noble metal composite aerogel fibers with a high specific surface area and high porosity by template casting. Yang et al. [33] successfully constructed cellulose acetate/polyacrylic acid (CA/PAA)-wrapped SF aerogel fibers toward textile thermal insulation using coaxial wet spinning. Unfortunately, these aerogel fibers generally have poor mechanical properties or complicated preparation processes. Therefore, it is still a huge challenge to prepare high-performance SF aerogel fibers with excellent mechanical properties and continuous production.

In this work, a green and simple method for preparing continuous aerogel fibers was developed. SF–agarose (AG) composite wet gel fibers were prepared using wet spinning of an SF–AG mixture obtained by mixing SF solution and AG solution and were then dried using supercritical carbon dioxide to obtain high-performance continuous SCAFs. In this process, the SF–AG wet gel fibers were formed rapidly as the AG had strong gel-forming properties, which enabled them to form gelation through physical crosslinking without an additional chemical crosslinking agent. Then, HSCAFs were obtained after silane modification using CVD. After the silanization, the HSCAF gel skeleton was covered with a rigid silica layer containing methyl groups. The rigid silica layer containing methyl groups enabled the HSCAFs to have a rigid and flexible gel skeleton. Most notably, the prepared HSCAFs exhibited outstanding radial compression and could recover their original volume after releasing the force at more than 60% strain. In addition, they displayed excellent thermal insulation performance at different temperatures, suggesting broader prospects for their use as new insulation materials for personal thermal management.

## 2. Results and Discussion

### 2.1. Design of HSCAFs

**Figure 1:**
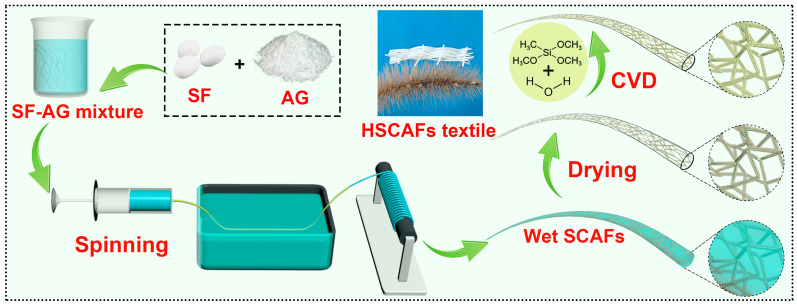
Schematic fabrication process for the HSCAFs.

A schematic illustration of the HSCAF preparation process, which involved wet-spinning, sol–gel, supercritical carbon dioxide (SC-CO_2_) drying, CVD, and weaving procedures, is shown in Figure 1. Briefly, SF–AG hydrogels were formed by wet spinning the SF–AG mixture in a water coagulation bath using an injection pump. Subsequently, the hydrogels were SC-CO_2_ dried to obtain 3D porous SCAFs. Due to the hydrophilic groups on the surface of the SF and AG molecules in the SCAFs, they were easily wetted when they came into contact with water. Finally, the as-prepared SCAFs were treated using CVD with methyltrimethoxysilane (MTMS) and H_2_O to obtain HSCAFs. The preparation processes for the agarose aerogel fibers (AAFs) and hydrophobic AAFs (HAAFs) were similar to those of the SCAFs and HSCAFs, except that the solution for wet spinning did not contain SF. The different concentrations of the SF and AG solutions used in the prepared HSCAFs and HAAFs are shown in Table 1. After silanization, the flexible SCAF gel skeleton was a uniformly coated rigid silica layer containing methyl groups, which endowed excellent radial elasticity.

### 2.2. Microstructure Characterization

**Figure 2:**
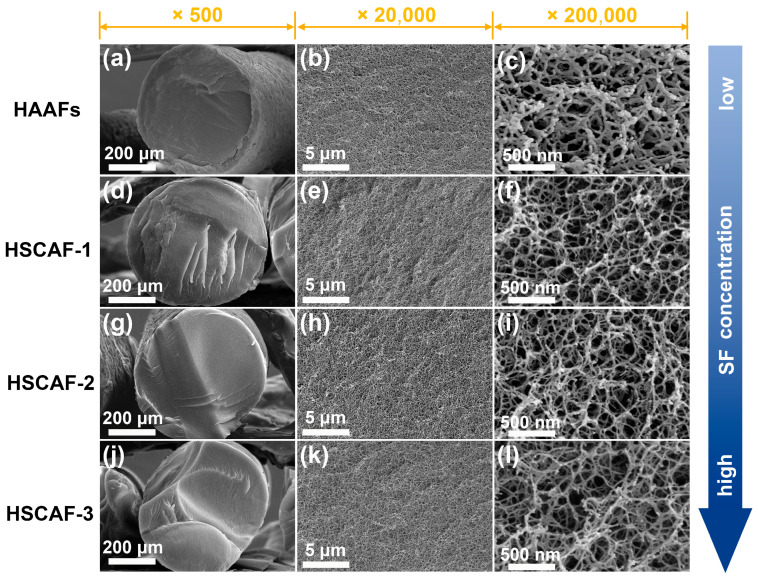
The SEM images of HAAFs (**a**–**c**), HSCAF-1 (**d**–**f**), HSCAF-2 (**g**–**i**), and HSCAF-3 (**j**–**l**) with different magnifications.

The microstructure of the HSCAFs was observed using scanning electron microscopy (SEM), as shown in Figure 2. The HSCAFs and HAAFs exhibited typical three-dimensional porous network structures and apparent diameters of approximately 0.6 mm at different concentrations of SF. By observing the cross-section of the HSCAFs (×500), it can be seen that the surfaces of the HSCAFs were relatively smooth, while the transverse surfaces of the HAAFs without SF were rough. This may be because the SFs in the HSCAFs contained a rigid β-sheet conformation, which increased their overall strength and reduced their flexibility, making it easier for them to present a flat and smooth sectional structure when they were broken by external forces. The gel skeleton size of the HSCAFs was relatively small compared with that of the HAAFs (×200,000), which may be attributed to the addition of SF to a certain extent hindering the aggregation of AG molecules during the gel process. Moreover, the three-dimensional network structure of the HSCAFs gradually became dense as the concentration of SF increased.

**Figure 3:**
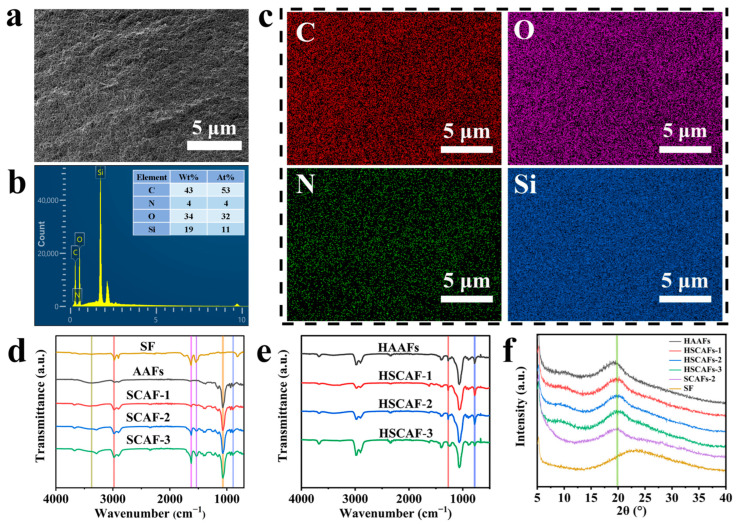
(**a**) The SEM images, (**b**) weight and element concentrations from the EDS, and (**c**) EDS elemental mapping images for the C, O, N, and Si elements of HSCAF-1. The FTIR spectra of (**d**) the SF, AAFs, SCAFs, (**e**) HAAFs and HSCAFs. (**f**) The XRD spectra of the SF, SCAFs and HSCAFs.

According to the microstructure of the HSCAFs (Figure 3a), energy-dispersive X-ray spectra (EDS) were captured to investigate the element distribution and weight concentration of the HSCAFs (Figure 3b,c). The EDS of the HSCAFs after silanization exhibited peaks corresponding to carbon, oxygen, silicon and nitrogen (Figure 3b). The appearance of a silicon peak and a mass concentration of 19% indicated that the silanization was successful. This was due to the fact that the HSCAF gel skeleton was uniformly coated with a layer of silica containing methyl during the silylation process. In addition, the nitrogen peak appeared before and after SF addition (Figure S1), indicating the successful combination of SF and AG in the HSCAFs. From the element-mapping distribution (Figure 3c), it can be seen that the distribution of Si and N in the HSCAFs was relatively uniform, which indicated that the SF and AG in the HSCAFs were evenly mixed via this synthesis method.

In the Fourier transform infrared (FTIR) spectrum of the AAFs, the characteristic peaks of AG at approximately 1070 cm^−1^, 890 cm^−1^, 2980 cm^−1^, and 3375 cm^−1^ indicated the C–O stretching vibration in the 3,6-dehydrated-β-galactose skeleton structure, the C–H stretching vibration peak in β-galactose, the C–H stretching vibration in the methylene groups, and the O–H contraction vibration peak (Figure 3d) [34,35,36]. With the addition of SF, two new characteristic peaks were found in the SCAFs, which were similar to those in the FTIR spectrum of SF. The characteristic peaks at 1625 cm^−1^ were amide I from the C=O stretching vibration of the peptide skeleton, which belonged to the absorption spectrum band of SF with irregular crimp conformation [37]. The characteristic peak at 1534 cm^−1^ was amide II, which corresponded to the β-sheet conformation [38]. Furthermore, the characteristic peaks of SF in the SCAFs were more significant with the increase in the SF content. Compared with the SCAFs, there were also two new characteristic peaks at 1272 cm^−1^ and 774 cm^−1^, corresponding to the Si–CH_3_ bending vibration and Si–O–Si stretching vibration in the FTIR spectrum of the HSCAFs modified by silanization (Figure 3e) [39]. This indicated that the silanization was successful. Moreover, the characteristic peaks of amide I and amide II of SF in the HSCAFs were not obvious compared with those of the SCAFs, which may illustrate that silane modification resulted in the HSCAF gel skeleton being uniformly coated with a silica layer containing methyl groups.

Figure 3f shows the X-ray diffraction (XRD) spectra of the SF, SCAF-2, HAAFs, and HSCAFs. An obvious diffraction peak can be seen at 19.3° for the HSCAF-1, representing the characteristic peaks of AG [40]. When SFs were added, the crystal diffraction peak of the HSCAFs gradually shifted to the right, and the peak position of the HSCAF-3 was 20.0°. This may be because the addition of SF reduced the AG aggregation, causing a change in the morphology of AG, which was consistent with the SEM analysis. Compared with the SCAF-2, the XRD pattern of the HSCAF-2 did not show a peak shift or the appearance of new peaks. This explained why the silanization of the SCAF-2 did not generate a new phase [2].

**Figure 4:**
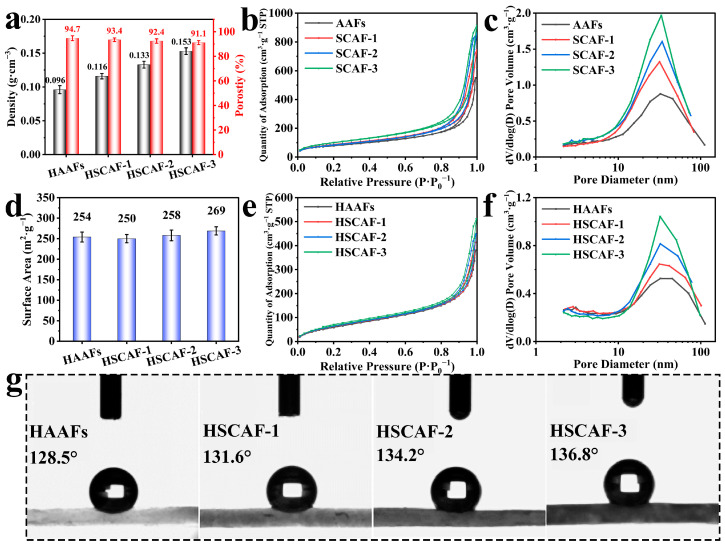
(**a**) Densities and porosities of the HAAFs and HSCAFs. (**b**) Nitrogen adsorption–desorption isotherms, and (**c**) BJH pore size distributions of the AAFs and SCAFs. (**d**) Specific surface areas, (**e**) nitrogen adsorption–desorption isotherms, and (**f**) BJH pore size distributions of the HAAFs and HSCAFs. (**g**) Water contact angles of the HAAFs and HSCAFs.

As the concentration of SFs increased, the densities of the HSCAFs gradually increased (Figure 4a), and their densities were higher than those of the SCAFs regardless of the concentration (Figure S2). This was due to the silica layer containing methyl groups coated on the HSCAF gel skeleton through silanization. The porosities of the HSCAFs gradually decreased with an increase in the SF concentration (Figure 4a), because the gel skeleton structure occupied more volume with an increase in the solid content.

### 2.3. Nitrogen Adsorption–Desorption Test and Wettability

The N_2_ adsorption–desorption isotherms of the AAFs, SCAFs, HAAFs and HSCAFs are shown in Figure 4b,e, revealing a type IV isotherm with an H1 hysteresis loop, proving that they had mesoporous structures [41]. The hysteresis loops of the HSCAFs became more obvious as the SF concentration increased. The Barrett–Joyner–Halenda (BJH) pore size distributions are shown in Figure 4c,f. The most probable pore size of the HSCAFs is approximately 20–30 nm, which again indicated that the HSCAFs had a mesoporous structure, and the distribution of the most probable pore size changed more significantly as the SF concentration increased. The adsorption capacity and pore size distribution of the HSCAFs modified with silane decreased compared to the SCAFs, which was attributed to a silica layer containing methyl groups uniformly coated on their gel skeleton. The specific surface areas of the aerogel fibers were determined using the BJH algorithm (Figure 4d). With the increase in the SF concentration, the specific surface area of the SCAFs increased (Figure S3), which can be attributed to the number of pores increasing with the increase in the solid content, and the most probable pore size distribution increased. After hydrophobic modification, the specific surface area of the HSCAFs decreased relatively, owing to the presence of SF making the fiber filaments on the gel skeleton thinner, and the thickness of the methyl-containing silica layer coated on the fiber filaments was fixed after the CVD, which resulted in a higher proportion of silicon oxide. Therefore, the specific surface area of the HSCAFs was relatively reduced.

The hydrophobicity of the HAAFs and HSCAFs was verified through water contact angle testing, as shown in Figure 4g. With the increase in the SF concentration, the water contact angles of the HSCAFs also increased, and their contact angles were more than 125°, indicating that the modified HSCAFs were hydrophobic. Combined with the SEM analysis, the gel skeleton of the HSCAFs gradually became thinner with the increase in the SF content, improving their surface roughness. At the same time, the silica layer coated with the methyl group on the gel skeleton was a hydrophobic layer, resulting in better hydrophobicity on a rougher surface.

### 2.4. Mechanical Properties

**Figure 5:**
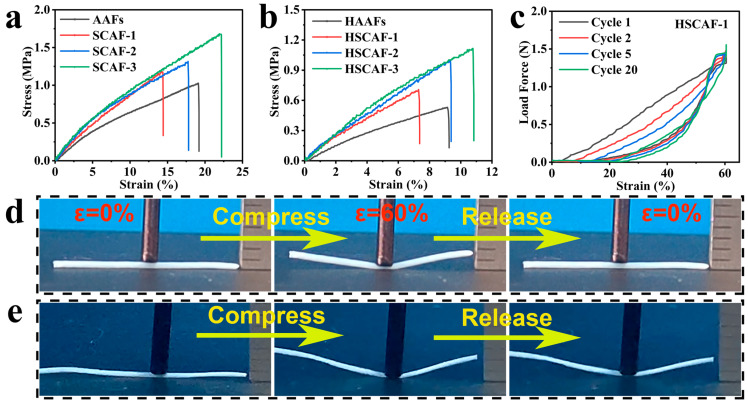
Tensile stress–strain curves for (**a**) the AAFs, SCAFs, (**b**) HAAFs and HSCAFs with different SF concentrations. (**c**) The cyclic compressive force–strain curves of the HSCAF-1 at 60% strain for 20 cycles. Digital images showing the compression and recovery of the (**d**) HSCAF-1 and (**e**) SCAF-1 at 60% strain.

The typical tensile stress–strain curves of the AAFs and SCAFs prepared with different concentrations of SF are shown in Figure 5a. As the concentration of SF increased, the tensile strength of the SCAFs gradually increased, indicating that the addition of SF had an enhancing effect on the AG matrix. This was because the SF contained a large number of random coil structures, and they had high aspect ratios and strong mechanical properties, effectively improving the tensile strength of the SCAFs. When a small amount of SF was added, the breaking elongation of the SCAFs was lower than that of the AAFs, making their ductility relatively lower owing to the existence of β-sheet conformation structures. In addition, the elongation at break of the SCAFs gradually increased with the increase in SF, reaching a maximum of 22%.

The tensile stress–strain curves of the HSCAFs prepared with different concentrations of SF are shown in Figure 5b. It can be seen that the tensile behavior of the HSCAFs after hydrophobic modification was basically the same as that of the SCAFs. However, their tensile strength and elongation at break decreased. This could be due to the generated rigid Si–O–Si structures on the HSCAF gel skeleton during hydrophobic modification, which prevented a relative slip between molecular chains. Moreover, as the concentration of SF increased, the tensile strength of the HSCAFs gradually increased.

For practical applications of aerogel fibers, they must withstand axial tension and radial compression, which is challenging for most aerogel fibers [27]. Compared with axial stretching, the radial compression of the aerogel fibers may be more important for practical wearing. When the ultra-porous aerogel fibers are subjected to external forces, their internal pores collapse or shrink, greatly reducing their thermal insulation performance. In order to evaluate the compression cycle of HSCAFs, it can be seen that the silane-modified HSCAF-1 almost returned to its original volume at 60% of the compression deformation (Figure 5d and Video S1). The cyclic compressive force–strain curve of the HSCAF-1 was further tested for 20 cycles, as shown in Figure 5c. The incompressible deformation of the HSCAF-1 during the first and second loading–unloading compression cycles at 60% strain was 8% and 12%, respectively. From the 5th loading to the 20th unloading, there was no significant change in the incompressible deformation (17–21%) of the HSCAF-1. This indicates that it experienced some loss of deformation after several previous compression deformations, and its structure tended to be stable when it was subjected to this further compression deformation. On the contrary, the SCAFs cannot rebound effectively owing to their flexible gel skeleton under an external force (Figure 5e and Video S2). This further exhibits that the rigid silica layer covered by the HSCAFs’ gel skeleton is essential to provide them with excellent radial elasticity.

### 2.5. Thermal Insulation Performance

**Figure 6:**
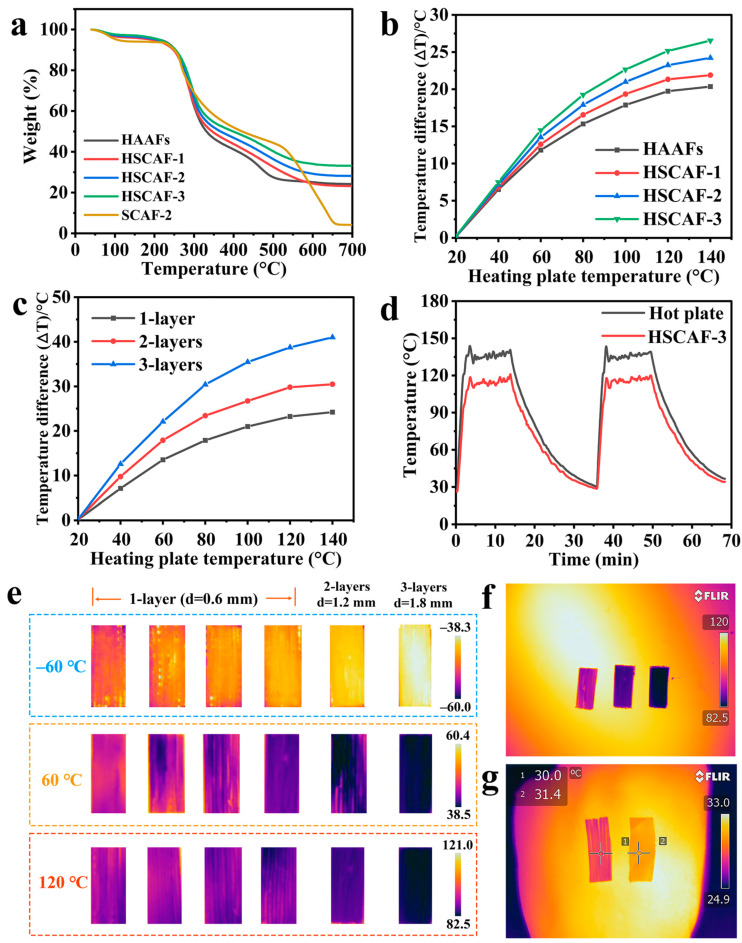
(**a**) Thermo-gravimetric analysis (TGA) curves for the HSCAFs. (**b**) Difference between the surface temperatures of the HSCAFs and hot plate. (**c**) Temperature difference between the HSCAF-3 surfaces and hot plate for the one- and three-layer thickness of the HSCAF-3. (**d**) Temperature–time curves for the HSCAF-3 and the hot plate. (**e**) Infrared photographs of the HSCAFs at different temperatures and different layers. (**f**) Infrared photo of the HSCAF-3 at different layer thicknesses. (**g**) Infrared image of the hand at room temperature, showing the thermal insulation capabilities of the HSCAF-3 and copper screen.

The thermal weight loss curves of the HSCAFs and SCAF-2 show three distinct stages of thermal weight loss, as shown in Figure 6a. The first stage of thermal weight loss at 40–120 °C was mainly caused by the evaporation of water in the HSCAFs. The second thermogravimetric stage was characterized by rapid thermal decomposition at 200 to 500 °C, owing to the destruction of the AG and SF molecular chains in the HSCAFs. Among them, side chain groups of amino acid residues were decomposed and peptide bonds were broken. Stabilization occurred at approximately 550 °C and mass loss no longer occurred, indicating the success of the silanization. After hydrophobic treatment, the residual mass was still more than 20%, which was because the surface of the HSCAF gel skeleton was covered with silica coating. In contrast, the thermal degradation of the SCAF-2 took place in three steps: 40–110 °C, 200–650 °C, and 650–700 °C. In the first stage (40–110 °C), degradation resulted from the evaporation-based loss of water from the pores. The main degradation of the SCAF-2 occurred from 200 to 650 °C in the second stage, originating from the combustion of the AG and SF skeleton in the SCAFs. In the third stage (650–700 °C), there was no significant weight loss, illustrating that the AG and SF were exhausted.

Several HSCAFs were arranged closely in one direction to form a single layer of pads approximately 0.6 mm thick and placed on the heating plate. The thermocouple was attached to the HSCAF surface and the heating plate, and the temperatures of the HSCAFs’ surface heated from 20 °C to 140 °C with a heating plate were recorded. The temperature differences of the HSCAFs with different concentrations of SF are shown in Figure 6b. When the heating plate was slowly heated from 20 °C to 140 °C, the temperature difference (|∆T|) of the HSCAFs also gradually increased as the concentration of SF increased. This was because the mesoporous structure of the HSCAFs became more obvious with the increase in the SF concentration, and the specific surface area also gradually increased, which effectively reduced the heat conduction and heat convection of the gas. At the same time, the gel skeleton became more compact and complex due to the increase in the solid content, which hindered solid heat conduction, resulting in better thermal insulation performance. Figure 6c showed the thermal insulation performance of the HSCAFs under different layers, and the results indicated that the superposition of layers can improve their thermal insulation performance. Figure 6d shows the precise values of the temperature changes on the surface of the hot plate and HSCAF-3 during the heating process. When the temperature of the hot plate increased from 30 °C to 135 °C, the surface temperature of the HSCAF-3 changed from 30 °C to approximately 115 °C. After the temperature stabilized, the |∆T| of both was approximately 20 °C. When the HSCAF-3 was heated again after a heating–cooling process, there was no significant change in |∆T|, indicating that the HSCAFs had a stable thermal insulation performance.

In order to more intuitively compare and display the thermal insulation performance of the HSCAFs, infrared images of the samples were taken using an infrared imager under different heat sources (Figure 6e). It can be seen that the thermal insulation effect of the HSCAFs gradually enhanced as the concentration of SF increased at different temperatures. In addition, the thermal insulation performance of the HSCAF-3 under different layers was compared, and it was found that the superposition of the surface layers can effectively improve the thermal insulation performance. Single-layer insulation can reach up to 24 °C at 120 °C, while three-layer insulation can reach 38 °C (Figure 6f). Single-layer insulation can reach a maximum of 12 °C under a cold source of −60 °C, and three-layer insulation can reach 22 °C. These results demonstrated that the HSCAFs had excellent thermal insulation performance. Moreover, the HSCAF-3 and a copper screen were used to conduct a heat insulation test on the back of the human hand (Figure 6g). At an ambient temperature of 24.9 °C, the |∆T| of the sample surface and the copper screen reached 2 °C, showing excellent thermal insulation performance.

## 3. Conclusions

In summary, SCAFs were prepared by mixing SF and AG through wet spinning, physical rapid gelation and SC-CO_2_ drying. In addition, the SCAFs were silanized using CVD to uniformly coat the gel skeleton with a silica layer containing methyl groups to obtain HSCAFs. This rigid silica layer endowed the HSCAFs with excellent radial elasticity and hydrophobicity (water contact angle of 136.8°). Moreover, the HSCAFs exhibited low density (≤0.153 g/cm^3^), a large specific surface area (≥254.0 m^2^/g), and high porosity (91.1–94.7%). Most notably, the HSCAFs demonstrated excellent insulation and thermal stability under both cold and hot conditions. In general, high-performance aerogel fibers are promising candidates for wearable materials, which can be used for personal thermal management.

## 4. Materials and Methods

### 4.1. Materials

The AG was purchased from Solaibao Technology Co., Ltd. (Beijing, China). The raw silk was supplied by Arongqi Dingsheng Cocoon Distribution Co., Ltd. (Hulun Buir, China). The anhydrous sodium carbonate (Na_2_CO_3_), lithium bromide (LiBr), and methyltrimethoxysilane (MTMS) were obtained from Aladdin Reagent Co., Ltd. (Shanghai, China). The anhydrous ethanol (C_2_H_5_OH) was purchased from China National Pharmaceutical Chemical Reagent Co., Ltd. (Shanghai, China). All the chemicals were used without further purification.

### 4.2. Preparation of SCAFs

The SF solution was prepared according to the method previously reported by Hyeon et al. [37]. The detailed process is described in the Supporting Information. A measure of 0.6g of AG was mixed with a certain amount of deionized water, and then the mixture was heated in a microwave until the AG was completely dissolved to obtain an AG solution. The SF–AG mixtures were prepared by mixing the SF solution and AG solution in a 70 °C oven. Firstly, a syringe (10 mL) was used to draw a certain amount of mixed solution, and then it was extruded by the injection pump at a constant rate (30 mL/h) during wet spinning. The needle was externally connected to a polytetrafluoroethylene hose, and the coagulation bath contained cold water (6 °C). This setup differs from the traditional wet-spinning process, where the coagulation bath typically plays a role in the chemical reactions required to promote gel formation. The speed of the jet pump had no effect on the thickness of the gel fibers. Then, the extruded SF–AG composite wet gel fibers were coiled and collected with a winder, fully replaced with absolute anhydrous ethanol, and then dried with SC-CO_2_ to obtain SCAFs. The preparation process for the AAFs was similar to that for the SCAFs, except that the wet-spinning solution did not contain SF. The different concentrations of the SF and AG solutions used in the mixture solution are shown in Table 1. The SCAFs were labeled as SCAF-1, SCAF-2, and SCAF-3.

### 4.3. Preparation of HSCAFs

CVD was used for the silane modification of the SCAFs and AAFs, where the SCAFs or AAFs and two vials containing MTMS (2 mL) and water (1 mL), respectively, were placed in a small dryer. A vacuum pump was used to vacuum the dryer to −0.08 MPa, and then putting the dryer into an oven at 50 °C for 2 h to obtain HSCAFs or HAAFs. The HSCAFs were labeled as HSCAF-1 to HSCAF-3 (Table 1) according to the concentration of SF and AG (corresponding to SCAF-1 to SCAF-3).

### 4.4. Characterization

The microstructure, functional groups (FTIR), density, porosity, pore size distribution, and specific surface area of the HSCAFs were measured, and their wettability, mechanical properties, thermal stability, and insulation performance were evaluated. The detailed characterization method can be found in the Supplementary Materials.

## Figures and Tables

**Table 1:** HSCAFs and HAAFs formed by the concentration of SF and AG.

Sample	HAAFs	HSCAF-1	HSCAF-2	HSCAF-3
SF (wt %)	0	0.5	1	1.5
AG (wt %)	2	2	2	2

## Data Availability

The original contributions presented in the study are included in the article, further inquiries can be directed to the corresponding authors.

## Supplementary Materials

The following supporting information can be downloaded at https://www.mdpi.com/article/10.3390/gels10040266/s1, Figure S1: EDS of the HAAFs; Figure S2: Densities and porosities of the AAFs and SCAFs; Figure S3: Specific surface areas of the AAFs and SCAFs; extraction and purification of SF; preparation of SF aerogels; characterization; Video S1: The compression and recovery of HSCAF-1; Video S2: The compression and recovery of SCAF-1.

## References

[1] Yang X., Jiang P.J., Xiao R., Fu R., Miao C.Q., Wen L.S., Song Q.Q., Liu Y.H., Yu H.Q., Gu J. (2022). Elastic Agarose Nanowire Aerogels for Oil-Water Separation and Thermal Insulation. ACS Appl. Nano Mater..

[2] Sai H., Fu R., Xing L., Xiang J., Li Z., Li F., Zhang T. (2015). Surface modification of bacterial cellulose aerogels’ web-like skeleton for oil/water separation. ACS Appl. Mater. Interfaces.

[3] Feng J., Su B.L., Xia H., Zhao S., Gao C., Wang L., Ogbeide O., Feng J., Hasan T. (2021). Printed aerogels: Chemistry, processing, and applications. Chem. Soc. Rev..

[4] Wang Z., E Y., Li J., Du T., Wang K., Yao X., Jiang J., Wang M., Yuan S. (2023). Sustainable bacterial cellulose-based composite aerogels with excellent flame retardant and heat insulation. Cellulose.

[5] Sai H., Wang M., Miao C., Song Q., Wang Y., Fu R., Wang Y., Ma L., Hao Y. (2021). Robust Silica-Bacterial Cellulose Composite Aerogel Fibers for Thermal Insulation Textile. Gels.

[6] Bian H., Duan S., Wu J., Fu Y., Yang W., Yao S., Zhang Z., Xiao H., Dai H., Hu C. (2022). Lignocellulosic nanofibril aerogel via gas phase coagulation and diisocyanate modification for solvent absorption. Carbohydr. Polym..

[7] Wu K., Fang Y., Wu H., Wan Y., Qian H., Jiang F., Chen S. (2021). Improving konjac glucomannan-based aerogels filtration properties by combining aerogel pieces in series with different pore size distributions. Int. J. Biol. Macromol..

[8] Lu T., Cui J., Qu Q., Wang Y., Zhang J., Xiong R., Ma W., Huang C. (2021). Multistructured Electrospun Nanofibers for Air Filtration: A Review. ACS Appl. Mater. Interfaces.

[9] Gia-Thien Ho T., Thao Truong D.P., Nguyen H.B., Long Do B., Dinh T.A., Ton-That P., Van Nguyen T.T., Ta Truong T.B., Ha Huynh K.P., Tri N. (2023). Green synthesized nano-silver/cellulose aerogel as a robust active and recyclable catalyst towards nitrophenol hydrogenation. Chem. Eng. J..

[10] Almeida C.M.R., Merillas B., Pontinha A.D.R. (2024). Trends on Aerogel-Based Biosensors for Medical Applications: An Overview. Int. J. Mol. Sci..

[11] Randall J.P., Meador M.A.B., Jana S.C. (2011). Tailoring Mechanical Properties of Aerogels for Aerospace Applications. ACS Appl. Mater. Interfaces.

[12] Tong Z., Zhang B., Yu H., Yan X., Xu H., Li X., Ji H. (2021). Si_3_N_4_ Nanofibrous Aerogel with In Situ Growth of SiO_x_ Coating and Nanowires for Oil/Water Separation and Thermal Insulation. ACS Appl. Mater. Interfaces.

[13] Teichner S.J., Nicolaon G.A., Vicarini M.A., Gardes G.E.E. (1976). Inorganic oxide aerogels. Adv. Colloid Interface Sci..

[14] Ren Q., Sun R., Feng D., Ru H., Wang W., Zhang C. (2022). Resorcinol formaldehyde hydrogel: Synthesis, polymerization, and application in ceramic gel-casting. Colloids Surf. A.

[15] Georgi M., Klemmed B., Benad A., Eychmüller A. (2019). A versatile ethanolic approach to metal aerogels (Pt, Pd, Au, Ag, Cu and Co). Mater. Chem. Front..

[16] Lu Z., Liu X., Wang T., Huang X., Dou J., Wu D., Yu J., Wu S., Chen X. (2023). S/N-codoped carbon nanotubes and reduced graphene oxide aerogel based supercapacitors working in a wide temperature range. J. Colloid Interface Sci..

[17] Rahmanian V., Pirzada T., Wang S., Khan S.A. (2021). Cellulose-Based Hybrid Aerogels: Strategies toward Design and Functionality. Adv. Mater..

[18] Yi L., Yang J., Fang X., Xia Y., Zhao L., Wu H., Guo S. (2020). Facile fabrication of wood-inspired aerogel from chitosan for efficient removal of oil from Water. J. Hazard. Mater..

[19] Chen X., Yang Y., Guan Y., Luo C., Bao M., Li Y. (2022). A solar-heated antibacterial sodium alginate aerogel for highly efficient cleanup of viscous oil spills. J. Colloid Interface Sci..

[20] Wang Z., Yang H., Li Y., Zheng X. (2020). Robust Silk Fibroin/Graphene Oxide Aerogel Fiber for Radiative Heating Textiles. ACS Appl. Mater. Interfaces.

[21] Ma X.Y.D., Zeng Z., Wang Z., Xu L., Zhang Y., Ang J.M., Wan M.P., Ng B.F., Lu X. (2022). Robust microhoneycomb-like nanofibrous aerogels derived from cellulose and lignin as highly efficient, low-resistant and anti-clogging air filters. J. Membr. Sci..

[22] Sheng Z., Liu Z., Hou Y., Jiang H., Li Y., Li G., Zhang X. (2023). The Rising Aerogel Fibers: Status, Challenges, and Opportunities. Adv. Sci..

[23] Yang S., Zhao C., Yang Y., Ren J., Ling S. (2023). The Fractal Network Structure of Silk Fibroin Molecules and Its Effect on Spinning of Silkworm Silk. ACS Nano.

[24] Yang H.W., Wang P., Yang Q.L., Wang D.F., Wang Y., Kuai L., Wang Z.Q. (2023). Superelastic and multifunctional fibroin aerogels from multiscale silk micro-nanofibrils exfoliated via deep eutectic solvent. Int. J. Biol. Macromol..

[25] Chen Y., Zhang C., Tao S., Chai H., Xu D., Li X., Qi H. (2023). High-performance smart cellulose nanohybrid aerogel fibers as a platform toward multifunctional textiles. Chem. Eng. J..

[26] Liu Z., Sheng Z., Bao Y., Cheng Q., Wang P.-X., Liu Z., Zhang X. (2023). Ionic Liquid Directed Spinning of Cellulose Aerogel Fibers with Superb Toughness for Weaved Thermal Insulation and Transient Impact Protection. ACS Nano.

[27] Wu M., Shao Z., Zhao N., Zhang R., Yuan G., Tian L., Zhang Z., Gao W., Bai H. (2023). Biomimetic, knittable aerogel fiber for thermal insulation textile. Science.

[28] Li M., Chen X., Li X., Dong J., Zhao X., Zhang Q. (2022). Controllable Strong and Ultralight Aramid Nanofiber-Based Aerogel Fibers for Thermal Insulation Applications. Adv. Fiber Mater..

[29] Xue T., Zhu C., Feng X., Wali Q., Fan W., Liu T. (2022). Polyimide Aerogel Fibers with Controllable Porous Microstructure for Super-Thermal Insulation Under Extreme Environments. Adv. Fiber Mater..

[30] He H., Wang Y., Liu J., Zhao Y., Jiang Q., Zhang X., Wang J., Wang H., Yu Z. (2023). Biomass based active-cum-passive aerogel heater with enhanced thermal insulation property derived from hollow cellulose kapok fiber for personal thermal management. Cellulose.

[31] Cui Y., Gong H., Wang Y., Li D., Bai H. (2018). A Thermally Insulating Textile Inspired by Polar Bear Hair. Adv. Mater..

[32] Mitropoulos A.N., Burpo F.J., Nguyen C.K., Nagelli E.A., Ryu M.Y., Wang J., Sims R.K., Woronowicz K., Wickiser J.K. (2019). Noble Metal Composite Porous Silk Fibroin Aerogel Fibers. Materials.

[33] Yang H., Wang Z., Liu Z., Cheng H., Li C. (2019). Continuous, Strong, Porous Silk Firoin-Based Aerogel Fibers toward Textile Thermal Insulation. Polymer.

[34] Wang J., Zhang W., Zhang C. (2019). Versatile fabrication of anisotropic and superhydrophobic aerogels for highly selective oil absorption. Carbon.

[35] Zarrintaj P., Bakhshandeh B., Rezaeian I., Heshmatian B., Ganjali M.R. (2017). A Novel Electroactive Agarose-Aniline Pentamer Platform as a Potential Candidate for Neural Tissue Engineering. Sci. Rep..

[36] Zhang Y., Fu X., Duan D., Xu J., Gao X. (2019). Preparation and characterization of agar, agarose, and agaropectin from the red alga *Ahnfeltia plicata*. J. Oceanol. Limnol..

[37] Kim H.J., Yang Y.J., Oh H.J., Kimura S., Wada M., Kim U.-J. (2017). Cellulose–silk fibroin hydrogels prepared in a lithium bromide aqueous solution. Cellulose.

[38] Lee K.G., Kweon H., Yeo J.H., Woo S.O., Lee J.H., Hwan Park Y. (2004). Structural and physical properties of silk fibroin/alginate blend sponges. J. Appl. Polym. Sci..

[39] Li N., Chen W., Chen G., Wan X., Tian J. (2018). Low-Cost, Sustainable, and Environmentally Sound Cellulose Absorbent with High Efficiency for Collecting Methane Bubbles from Seawater. ACS Sustain. Chem. Eng..

[40] Chhatbar M.U., Godiya C., Siddhanta A.K. (2012). Functional modification of agarose: A facile synthesis of an agarose-saccharate derivative. Carbohydr. Polym..

[41] Gao R., Xiao S., Gan W., Liu Q., Amer H., Rosenau T., Li J., Lu Y. (2018). Mussel adhesive-inspired design of superhydrophobic nanofibrillated cellulose aerogels for oil/water separation. ACS Sustain. Chem. Eng..

