# Supplementary material for: Hydrophobic Silk Fibroin–Agarose Composite Aerogel Fibers with Elasticity for Thermal Insulation Applications
Source: Gels. 2024 Apr 15;10(4):266. doi: 10.3390/gels10040266 (PMC11049485; doi:10.3390/gels10040266)
Supplement: Supplementary file 1 [file gels-10-00266-s001.zip › Revised Supporting Information.pdf]

gels

MDPI

Supplementary

Hydrophobic Silk Fibroin–Agarose Composite Aerogel Fibers with Elasticity for Thermal Insulation Applications

Yuxiang Du[1,2,†], Pengjie Jiang [1,2,†], Xin Yang [1,2], Rui Fu [1,2,*], Lipeng Liu [1,2], Changqing Miao [1,2], Yaxiong Wang [1,2] and Huazheng Sai [1,2,*]

[1] School of Chemistry and Chemical Engineering, Inner Mongolia University of Science and Technology, Baotou 014010, China; duyuxiang5520@163.com (Y.D.); jpj1692787089@163.com (P.J.); yangxin975@163.com (X.Y.); li-pengliu1998@163.com (L.L.); qingmc@163.com (C.M.); wangyaxiong2021@126.com (Y.W.)

[2] Aerogel Functional Nanomaterials Laboratory, Inner Mongolia University of Science and Technology, Baotou 014010, China

* Correspondence: furui14@mails.ucas.edu.cn (R.F.); shz15@tsinghua.org.cn (H.S.)

Citation: Du, Y.; Jiang, P.; Yang, X.; Fu, R.; Liu, L.; Miao, C.; Wang, Y.; Sai, H. Hydrophobic Silk Fibroin–Agarose Composite Aerogel Fibers with Elasticity for Thermal Insulation Applications. *Gels* **2024**, *10*, 266. https://doi.org/10.3390/gels10040266

Academic Editor: Miguel Sanchez-Soto

Received: 23 March 2024
Revised: 8 April 2024
Accepted: 12 April 2024
Published: 15 April 2024

Copyright: © 2024 by the authors. Licensee MDPI, Basel, Switzerland. This article is an open access article distributed under the terms and conditions of the Creative Commons Attribution (CC BY) license (https://creativecommons.org/licenses/by/4.0/).

1. Supplementary Video

Video S1: the compression and recovery of HSCAF-1; Video S2: the compression and recovery of SCAF-1.

2. Materials and Methods

2.1 Extraction and purification of SF

First, the cocoons were cut open to remove the chrysalis and impurities in the cocoon, and peeled off the inner membrane connected with the cocoons. Then, cocoons were boiled in 0.02 M Na_2CO_3 solution (w/w, 1:50) for 20 min and rinsed thoroughly with deionized water to remove the glue-like sericin proteins and wax. The purified SF was then dissolved in a 9.3 M LiBr aqueous solution (w/w, 1:10) at 65 °C for 4 h. This solution was dialyzed in deionized water using a cellulose dialysis membrane (molecular weight cutoff, 3000–3500) for 3 days.

2.2 Preparation of SF aerogels

The processed SF solution was frozen in a cold trap at −60 °C for 3 h, followed by freeze-drying in a vacuum (<12 Pa) for 72 h to obtain SF aerogels.

3. Characterization

3.1 Morphology and Nanostructure

The micromorphology of HAAFs and HSCAFs was analyzed by SEM using Thermo Scientific Apreo 2C (USA) at accelerating voltage of 10 kV and a working distance of 10 mm. In order to obtain a complete cross-section of the aerogel fiber, the fiber was broken in the state of wet gel, and then the complete cross-section of the sample to be measured was obtained by SC-CO_2 drying and CVD. Samples were stuck on the sample holder with a carbon pad and coated with 7.5 nm of platinum.

3.2 Energy-Dispersive X-ray Spectra (EDS)

The relative elements content of the HSCAFs and HAAFs were determined by EDS. The test conditions were nearly the same as those used for SEM characterization, yet the samples were thicker.

3.3 Fourier Transform Infrared (FTIR) Spectroscopy Analysis

FTIR infrared spectroscopy analysis was performed to evaluate the chemical changes the samples. The FTIR spectra of the samples were obtained by a PerkinElmer (Spectrum 3, American). The scan range of FTIR spectroscopy was 500-4000 cm^{-1}.

3.4 Density

The density of samples was determined by measuring the weight and volume of each individual sample. The weight of each aerogel fiber was measured using an analytical balance (readability 0.0001 g, Sartorius). The dimensions of each aerogel fiber were measured by a digital caliper. Five fibers were used for density determination for each sample and averaged.

3.5 Porosity

The porosity (P) of the HSCAFs and SCAFs was calculated according to Equation S1, where ρ, ρ_s and ρ_a are the bulk density of HSCAFs or SCAFs, the skeleton densities of pure SF and AG; ω_s and ω_a were the mass fraction of SF and AG, respectively. Herein, the ρ_s and ρ_a were fixed at 1.46 g/cm^3 and 1.80 g/cm^3.

$$P\ (\%) = \left(1 - \frac{\rho}{\omega_s\rho_s + \omega_a\rho_a}\right) \times 100\% \quad (S1)$$

The porosity (P) of the AAFs and HAAFs was calculated according to Equation S2, where ρ, and ρ_a are the bulk density of AAFs or HAAFs, the skeleton densities of pure AG, respectively. Herein, the ρ_a was fixed at 1.8 g/cm^3.

$$P\ (\%) = \left(1 - \frac{\rho}{\rho_a}\right) \times 100\% \quad (S2)$$

3.6 Nitrogen Physisorption Measurements

The samples (~30 mg) were measured the nitrogen adsorption and desorption isotherm, pore size distribution, and specific surface area at 77 K by physical adsorption instrument (ASAP 2460, Shanghai). The samples were first outgassed at 130 °C for 5 h before measuring. The amount of N_2 absorbed at various relative vapor pressures (nine points where the pressure is $0.05 < p/p_0 < 0.3$, the cross-sectional area of nitrogen molecules is calculated as 0.162 nm^2) was used to determine the surface area by Brunauer-Emmett-Teller (BET) analysis method. The pore size distribution of the relevant samples was obtained in the adsorption-desorption curves by the Barrett-Joyner-Halenda (BJH) algorithm.

3.7 Wettability test

The contact angle of HSCAFs was tested by using a static drip contact angle measuring instrument (SZ-CAMC31, Shanghai XuanZhun Instrument Co., Ltd.), and the test method was to determine the static contact angle by the seat drop method. A droplet of deionized water suspension (the volume of liquid in suspension is 2 μL) was constructed using a micro-syringe, and the fibers were straightened using a fixture and placed on the sample stage. The contact angles were measured at different points of the fibers and averaged, and the number of measurement points was at least 5, and the error is within ±0.5°. By raising the sample stage and adjusting it from side to side to bring the droplets into slow contact with the surface of the sample to be tested, the image was captured by an image sensor, and a baseline was constructed and then analyzed using software for contact angle analysis.

3.8 Mechanical Properties

The mechanical properties of the aerogel fibers were tested by an electronic universal testing machine (HD-B609B-S, Guangdong, China), in the tensile mode. The strain rate was 2 mm/min for the tensile tests. All the test samples were 2 cm. The compressive rate was set as 6 mm/min. Each set of tensile strength test results are collected from at least five samples to obtain reliable values. In order to obtain the samples required for testing mechanical properties, HSCAFs and HAAFs were obtained by SC-CO_2 drying and CVD after cracking fibers with a growth of approximately 2 cm in the preparation of wet gel fibers.

3.9 Thermal Stability

The thermal degradation behavior of the HSCAFs and SCAFs in air atmosphere was analyzed and investigated by thermogravimetry analyzer (TGA, STA449 F3). A sample weight of approximately 2 mg was used and heated from 30 to 700 °C at a heating rate of 10 K/min in a ceramic pan.

3.10 Thermal Insulation Measurement

The infrared imaging was gained by a thermal infrared camera (FLIR T620, USA). The camera was operated at a distance of about 10-30 cm. The samples were put on the heating plate or aluminum plate of dry ice. The samples were tested after 10 min of remaining at a constant surface temperature.

3.11 X-ray diffraction (XRD)

XRD analysis is used to determine the crystallinity of HSCAFs, and the samples were tested using an Empyream type powder X-ray diffractometer. The working current was 400 mA, the working voltage was 45 kV, and the diffraction intensity curve of the diffraction angle 2θ was between 5 and 40 under the condition of Cu target.

4. EDS of HAAFs

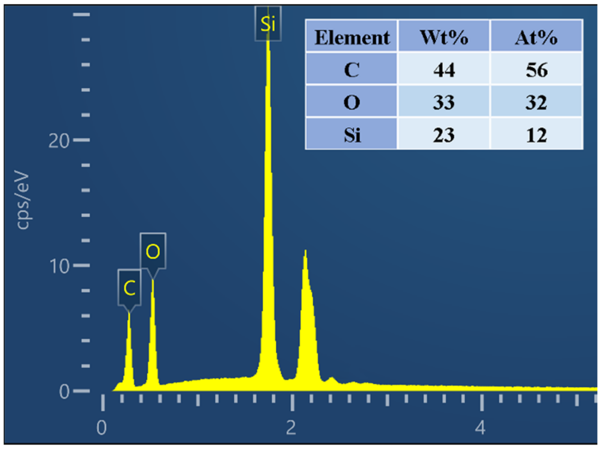

Figure S1. Weight concentration from EDS for the HAAFs.

5. Densities and porosities of AAFs and SCAFs

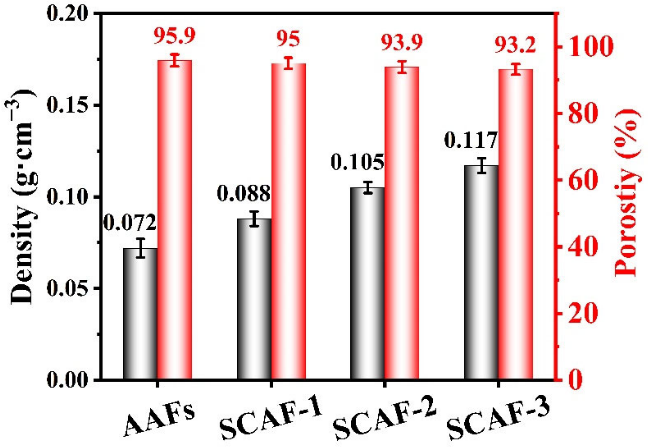

Figure S2. Densities and porosities of AAFs and SCAFs.

6. Specific surface areas of the AAFs and SCAFs

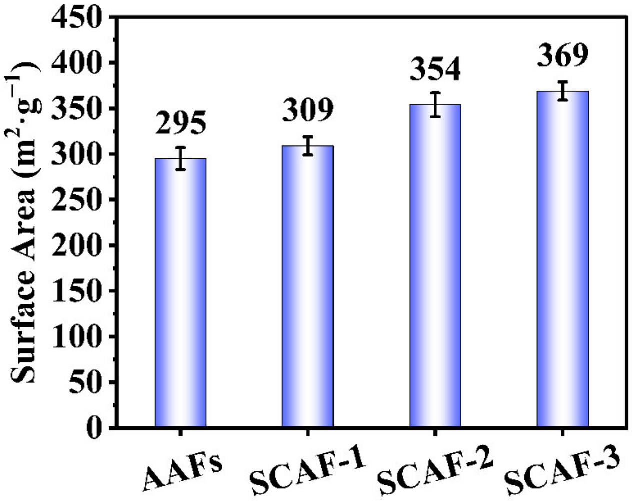

Figure S3. Specific surface areas of the AAFs and SCAFs.

Disclaimer/Publisher's Note: The statements, opinions and data contained in all publications are solely those of the individual author(s) and contributor(s) and not of MDPI and/or the editor(s). MDPI and/or the editor(s) disclaim responsibility for any injury to people or property resulting from any ideas, methods, instructions or products referred to in the content.
